# Estimate the severity of acute ischemic stroke by optic nerve sheath ultrasound

**DOI:** 10.1186/s13089-025-00465-x

**Published:** 2025-12-08

**Authors:** Sara Esmaeili, Farzan Vahedifard, Fatemeh Ebrahimi, Hossein Nazarian, Arian Shahidi, Zahra Mirzaasgari, Mohammadreza Maghsoudi

**Affiliations:** 1 Department of Neurology, Iran University of Medical Sciences, Tehran, Iran; 2 Cellular and Molecular Research Centre, Iran University of Medical Sciences, Tehran, Iran; 3 Student Research Committee, Iran University of Medical Sciences, Tehran, Iran; 4 Department of Neurology, Firoozgar Hospital, Iran University of Medical Sciences, Tehran, Iran; 5 College of Education and Human Sciences, Oklahoma State University, Stillwater, OK USA; 6 Faculty of Medicine, Amir-al-Momenin Hospital, Tehran Medical Sciences, Islamic Azad University, Tehran, Iran; 7 Department of Emergency Medicine, School of Medicine, Shahid Rajaei Hospital, Shahid Madani Hospital, Alborz University of Medical Sciences, Karaj, Iran

**Keywords:** Ultrasound, Optic nerve sheath diameter, Stroke, Severity, ICP

## Abstract

**Background:**

Timely diagnosis of acute ischemic stroke can aid optimal treatment. Optic nerve sheath diameter (ONSD) can determine increased intracranial pressure (ICP) in such cases. The purpose of this study is to determine the value of ONSD in estimating the severity of acute ischemic stroke.

**Methods:**

Patients with acute ischemic stroke who were referred to a stroke center were studied. The ONSD of both the right and left sides was measured by ultrasound on the day of admission. Ischemic stroke severity was determined based on the NIHSS.

**Results:**

A strong correlation was found between increased right and left ONSDs and severity of ischemic stroke determined by the initial NIHSS score. Based on ROC curve (receiver operating characteristic curve) analysis, both cut points of 5.65 mm for right ONSD (with 100% sensitivity of and 86% specificity) and 5.75 mm for left ONSD (with a 100% Sensitivity and 88% specificity) were able to predict severe stroke. The value of the right ONSD (Area Under the Curve = 0.959) and the left ONSD (Area Under the Curve = 0.942) indicated a strong predictive value.

**Conclusions:**

Ultrasound as a feasible and non-invasive modality might play a role in determining the severity of an acute ischemic stroke, and could be considered a promising first-line decision making tool.

## Introduction

Stroke is a major global health problem and a medical emergency that often results in disability and death [1]. Neuronal damage due to ischemia activates a pathologic cascade that causes brain edema which, in turn, results in raised intracranial pressure [2]. Cerebral edema is the most important cause of early death in stroke patients [3]. Timely diagnosis and treatment are critical factors determining the outcome of an acute stroke [4].

In areas around the ischemic region, known as ‘the penumbra region’, cell injury is reversible and collateral blood flow can effectively reduce the ischemic effect [4, 5]. Maintenance of cerebral perfusion pressure (CPP) is one of the most effective methods to protect the penumbra region from ischemia [5]. Since there is an inverse relationship between intracranial pressure (ICP) and CPP, high ICP exacerbates the ischemia and worsens this condition [6]. Thus, it might be an indirect index of irreversible neurologic damage and could be associated with mortality, morbidity, and poor consequences [7]. Prompt diagnosis and treatment of raised ICP may assist patients in survival and recovery, and high ICP is associated with poor outcomes in any brain injury. In a review, the mortality was 18.4% for patients with ICP less than 20 mm Hg, and 55.6% for those with ICP more than 40 mm Hg [8–10]. The normal ONSD range seems to be between 2 and 4.35 mm, but this is increased to 4.55 to 7.66 mm in instances of increased ICP [11].

Methods of measuring ICP are classified into either invasive or non-invasive forms. Traditionally, non-invasive methods include imaging methods like computerized tomography (CT), magnetic resonance imaging (MRI), MRI angiography, and recently Transcranial Doppler ultrasonography (USG) [12].

A relatively novel approach is the ultrasonic evaluation of the optic nerve sheath, which might provide a direct connection between increased ICP, due to trauma, and a rise in optic nerve sheath diameter [13]. Ultrasound is a simple, reliable, non-invasive diagnostic method that is very useful in patients with probable increased ICP as a result of trauma. Ultrasonography is considered a promising and efficient tool available in emergency departments that can be used as a first-line surrogate method for clinicians [14]. If ultrasound techniques reliably detect the severity of acute ischemic stroke due to increased ICP, the treatment can be rapidly started even in places where CT scan or MRI are not available. It is hypothesized that optic nerve sheath diameter is also an indicator of elevated ICP rising [15].

The purpose of this study is to determine optic nerve sheath diameter (ONSD) as a radiological marker of stroke severity.

## Materials and methods

### Study design

In an analytical cross-sectional study, patients with acute ischemic stroke, who were admitted to a major university hospital, from September 2019, to January 2020 were recruited to the study.

The inclusion criteria were patients with ischemic stroke during the past 6 h diagnosed by corresponding signs and symptoms along with appropriate imaging, patients aged over 18 years, and those with a maximum door-to-imaging time of 1 h. Patients with a current or past history of hydrocephalus or any other condition associated with an increase in ICP such as tumors, pseudotumor cerebri, infections, tumors, stroke, aneurysm, epilepsy, seizures, initial Brain CT scan or MRI indicative of increased ICP, history of any brain shunt or cranial surgery and those with a known history of ophthalmic diseases (e.g. glaucoma) were excluded from the study. The history of the patients was obtained from family members, medical records, and clinical screening.

On admission, data including age, gender, and type of ischemic stroke based on TOAST classification [16] (1. cardioembolic, 2. large vessels occlusion, 3. small vessels atherosclerosis, 4. stroke of determined etiology 5. stroke of undetermined etiology), and the brain hemisphere affected were recorded. The severity of stroke on admission was determined using by NIHSS(National Institutes of Health Stroke Scale).

### Optic nerve sheath ultrasound

Within 1 h of admission, ONSD measurement of both eyes was performed by ultrasound by an experienced sonographer specializing in ocular ultrasound. To measure the ONSD, patients were settled in a supine position and 20° to the horizontal, and a 6–13 MHz linear-array probe (L25x, Sonosite M-Turbo, USA) was placed on the orbit. Preventing pressure on the eye, the probe was placed only on the temporal area of the eyelid, not the eyelid itself. The depth of the field was 4 cm, and the mode was 2-dimensional.

The optic nerve was identified as a hypoechoic band emerging from the posterior aspect of the globe. The ONSD was then measured perpendicular to the optic nerve axis at a depth of 3 mm behind the inner surface of the posterior sclera.

**Fig. 1:**
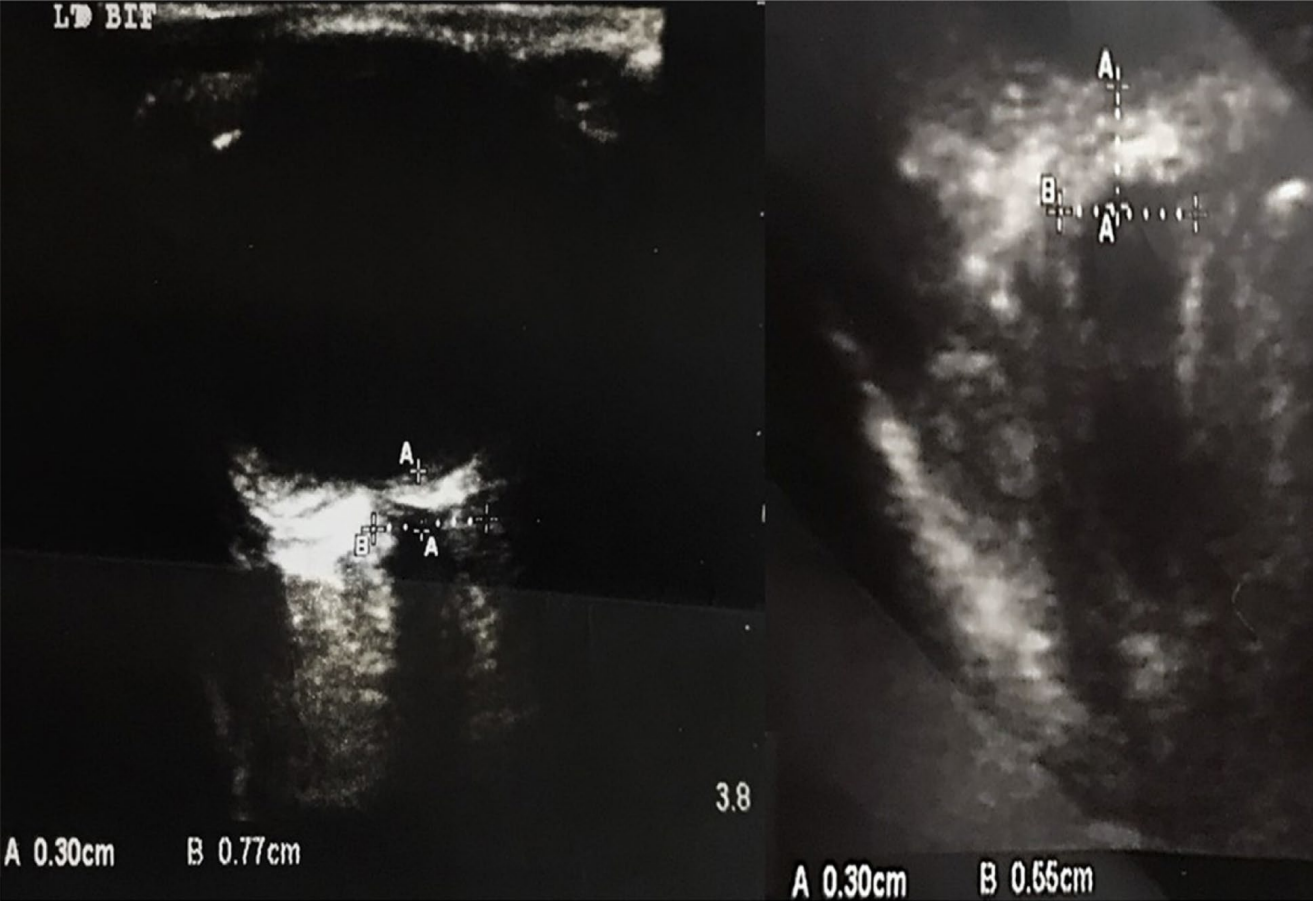
Ultrasound of optic nerve sheath using an electronic caliper along the axis of the optic nerve

The OSND was measured through an electronic caliper and vertical axis to the optic nerve (Fig. 1). Two measurements were made: 1—sagittal plane and 2—transverse plane (via clockwise rotation) [1]. 3—measurements were obtained for each eye and an average was recorded.

### Data analysis

Results were presented via mean and standard deviations (mean ± SD) for quantitative variables and as percentiles for qualitative variables. A comparison between quantitative variables was performed through t-testing. A chi-square test was used to compare the qualitative variables. The area under the ROC curve was used to determine the value of ONSD in predicting the severity of stroke. Data were analyzed by SPSS version 26 and SAS version 9.1. The significance level of 0.05 was utilized.

To assess the prognostic value of ONSD, the NIHSS was also recorded at the time of patient discharge. The change in NIHSS (ΔNIHSS) was calculated as the admission NIHSS score minus the discharge NIHSS score, where a positive value indicates clinical improvement. Pearson’s correlation coefficient was used to evaluate the relationship between baseline ONSD measurements and ΔNIHSS.

### Ethical considerations

Competent patients or substitute decision-makers(for non-competent patients) were informed of the purpose of the study and how the study would be conducted. They were assured of the safety and privacy of the procedure (information would be shared only with the project manager), as well as about getting routine treatment even if opting not to participate in the study. Furthermore, patients participating in the study were not subject to medical charges or fees. All stages of the project were approved by the Research Ethics Committee (Code: 1396.8911215369).

## Results

### Basic characteristics

A total of 98 patients with acute ischemic stroke were presented to the center and 60 of those met the inclusion criteria of the study and were included. The mean age of patients was 62.72 years, ranging from 31 to 80 years. The majority of patients were male (56.7%) and half of the strokes were cardioembolic. In terms of hemisphere involvement, right and the left hemispheres were almost equally affected (48.3% and 51.7%, respectively, P-value > 0.05). The mean NIHSS score was 6.93 on admission. Data of basic characteristics are depicted in Table 1.

**Table 1:** Background characteristics of patients with ischemic stroke

	Values	
Mean age (mean ± SD)	62.72 years ± 12.06^1^	
Gender distribution	Male	34 cases (56.7%)^2^
	Female	26 cases (43.3%)
Stroke type	Cardioembolic	30 cases (50.0%)
	Large vessels occlusion	10 cases (16.7%)
	Small vessels atherosclerosis	18 cases (30.0%)
	Others	2 cases (3.3%)
Involved hemisphere	Right	29 cases (48.3%)
	Left	31 cases (51.7%)
Stroke severity	Mild stroke (NIHSS ≤ 5)	34 cases (56.7%)
	Moderate stroke (5 < NIHSS ≤ 13)	16 cases (26.7%)
	Severe stroke (NIHSS > 13)	10 cases (16.7%)
NHISS score on admission (mean ± SD)	6.93 ± 4.50^1^	

^1^Quantitative variables are presented in mean ± SD

^2^Qualitative variables are presented in percentiles

### ONSD

The mean right ONSD on admission was 5.42 ± 0.41 mm, and the mean left ONSD was 5.43 ± 0.47 mm which showed no significant difference (P-value = 0.206).

### Correlations

**Fig. 2:**
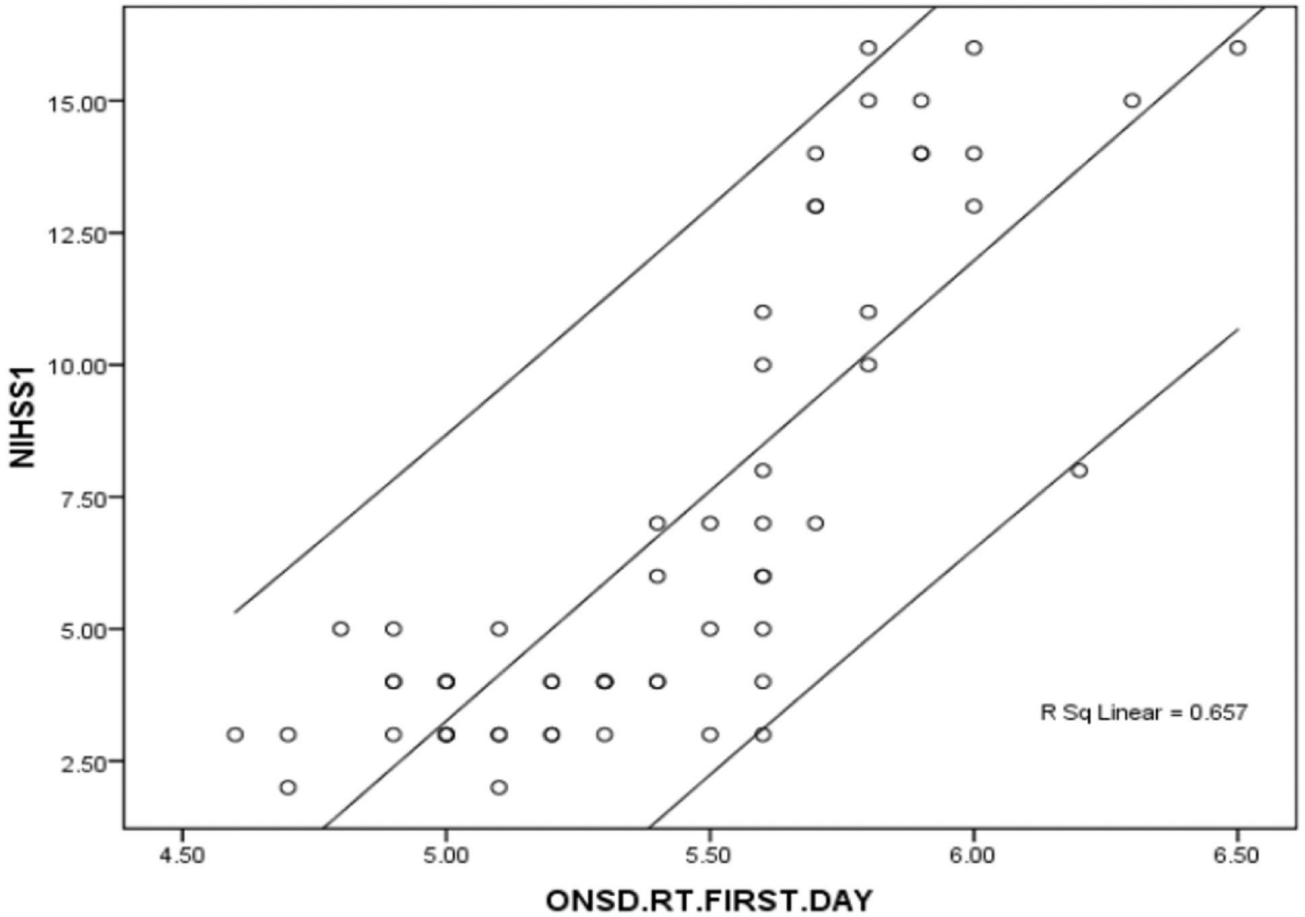
Diagram shows strong correlation between NIHSS score and right ONSD on admission

**Fig. 3:**
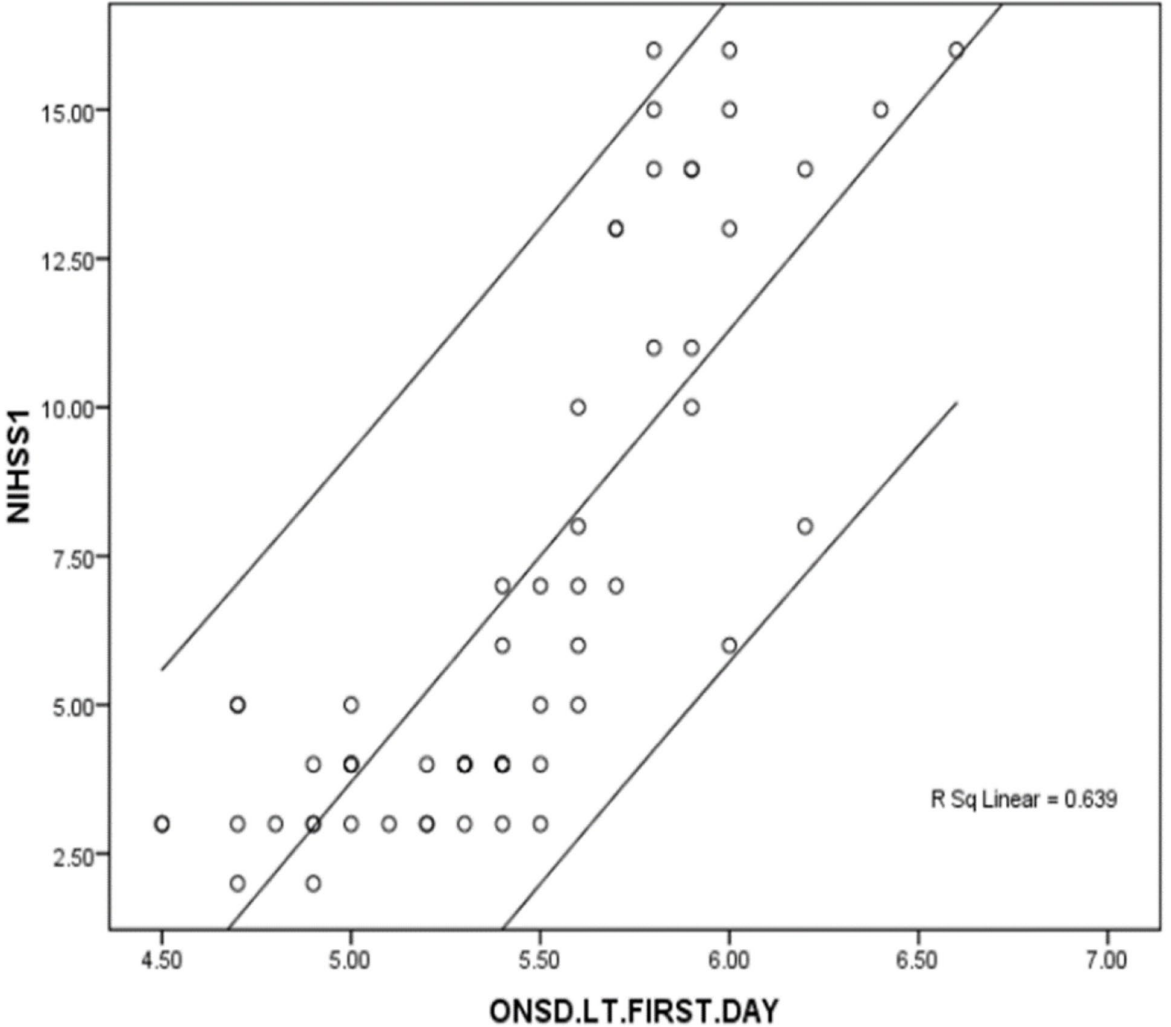
Diagram shows strong correlation between NIHSS score and left ONSD on admission

There was a significant correlation between the severity of ischemic stroke based on initial NIHSS and right ONSD (correlation coefficient 0.810, P value < 0.001), and as well as left ONSD values (correlation coefficient 0.799, P value < 0.001) (Figs. 2 and 3). The correlation between ONSD and stroke-related factors is summarized in Table 2. The mean right and left ONSD was significantly higher in females compared to men (P-value of 0.037 and 0.038 respectively). There was no correlation between age of patents and mean right ONSD (correlation coefficient = 0.174, P-value = 0.184) or mean left ONSD (correlation coefficient = 0.120, P-value = 0.360). The mean right and left ONSD at baseline were not significantly different in patients with different types of stroke. Likewise, the mean right and left ONSD at baseline showed no difference (p-value > 0.05) in patients with left- or right- hemispheric ischemic strokes. (p-value > 0.05)

**Table 2:** Correlation between ONSD on admission and baseline characteristics

Index	Right ONSD	Left ONSD
Gender		
Male	5.32 ± 0.40	5.31 ± 0.46
Female	5.55 ± 0.42	5.57 ± 0.46
P-value	0.037	0.038
Stroke type		
Cardioembolic	5.36 ± 0.47	5.35 ± 0.53
Large vessels occlusion	5.67 ± 0.33	5.71 ± 0.35
Small vessels atherosclerosis	5.39 ± 0.30	5.38 ± 0.34
Other types	5.45 ± 0.78	5.55 ± 0.92
P-value	0.288	0.195
Involved hemisphere		
Right	5.36 ± 0.36	5.31 ± 0.43
Left	5.47 ± 0.46	5.52 ± 0.50
P-value	0.320	0.079

### ROC curve

Based on the ROC curve analysis, the values ​​of right ONSD (Area under a Curve level of 0.959) and left ONSD (Area under a Curve level of 0.942) were strongly predictive of severe ischemic stroke (P-value < 0.001) (Table 3).

**Table 3:** Statistical significance testing of the area under ROC curve

Area under curve					
Test result Variable(s)	AUC	Std. error	Asymptotic Sig.	95% Confidence interval	
				Lower bound	Upper bound
ONSD.RT	0.959	0.024	0.000	0.912	1.006
ONSD.LT	0.942	0.029	0.000	0.885	0.999

**Fig. 4:**
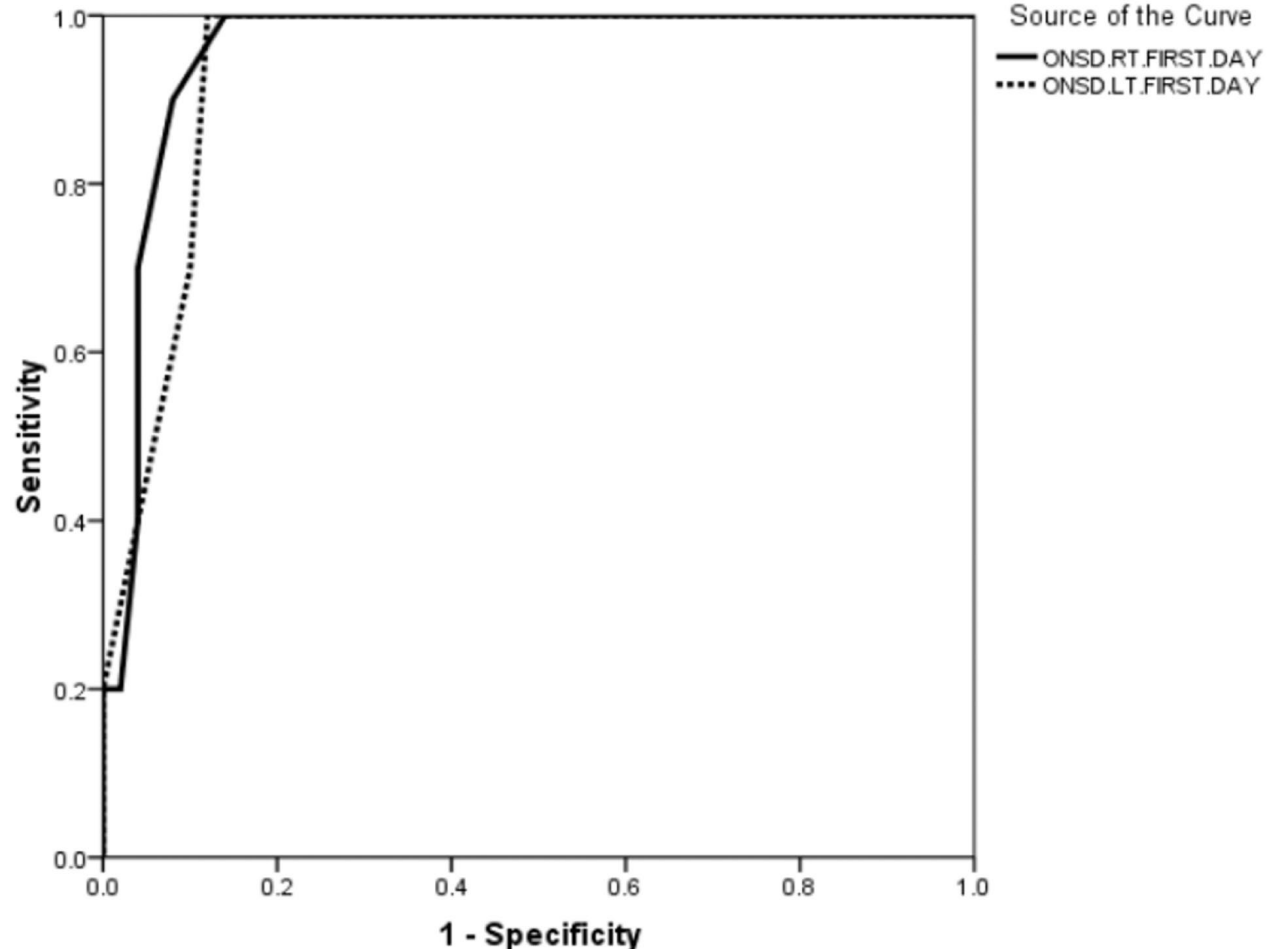
Area under ROC curve (AUC) in determining right and left ONCD for predicting severity of acute stroke on the day of admission

Cutoff points of 5.65 mm for right ONSD (100% sensitivity and 86% specificity) and 5.75 mm for left ONSD (100% sensitivity and 88% specificity) were able to predict severe stroke strongly. They were considered optimal cutoff points (Table 3; Fig. 4).

### ONSD as a prognostic indicator

The relationship between baseline ONSD and clinical improvement (ΔNIHSS) was analyzed. A strong negative correlation was observed between the admission ONSD and ΔNIHSS. Specifically, the right ONSD correlated with ΔNIHSS (*r* = − 0.72, *p* < 0.001), and the left ONSD correlated with ΔNIHSS (*r* = − 0.69, *p* < 0.001). This indicates that a higher ONSD at presentation is associated with less clinical improvement during hospitalization.

## Discussion

The results of this study indicate a predictive value in the ultrasonographic evaluation of optic nerve sheath diameter (ONSD) for assessing the severity of acute ischemic stroke. Previous studies have evaluated the utilization of ONSD in predicting elevated ICP, as well as predicting the occurrence of hemorrhagic events [1]. Nevertheless, as a novel idea, the occurrence of ischemic brain injury and its severity might be predictable with increasing ONSD.

The results of the study showed that the ONSD was a strong and reliable tool for predicting high NIHSS scores as well as overall stroke severity. Additionally, by identifying the best cut-off points for ONSD values ​​with high sensitivity and specificity, our study allowed for a distinction between severe and mild to moderate ischemia with high significance.

To explain the results, it should be emphasized that the optic nerve sheath extends over the dorsum which also covers the optic nerve to the back of the eye [2]. Studies have shown that changes in ONSD reflect changes in ICP. This is related to the flow of subarachnoid fluid into the optic nerve sheath. It is directly associated with the extension of this fluid to the intracranial segments of the optic nerve, which is covered by the leptomeningeal sheath as parts of the central nervous system [3]. Subarachnoid fluid enters the anterior segment behind the cavity of the eye, and when CP increases, the cerebrospinal fluid (CSF) moves forward to the subarachnoid, between the nerve sheath and the nerve itself, and extends the dural covering. These changes will be most pronounced in the anterior portion of the nerve sheath, behind the eyeball. Thus, in the physiological state, ONSD changes are associated with increased ICP [5]. Consequently, in all types of hemorrhagic strokes or in events that lead to increased ICP, a concomitant increase in ONSD is expected [6]. Yet, there are many questions about the significant increase in ONSD in a variety of cerebral ischemic events. The exact pathophysiology in cases of ischemic stroke remains unclear so far.

Few cross-sectional studies have evaluated the relationship between increased ONSD and the severity of ischemic events in the brain. Gökcen et al. [17], along with our results, indicated that the amount of ONSD in the ischemic stroke group was significantly higher than in the healthy controls, especially in anterior cerebral artery infarction. In the Hedna et al. study [18] ONSD levels in patients with ischemic stroke were directly related to the risk of death, thus, suggesting it is a predictor of mortality in these patients. However, they predicted a cut-off point of 6.2 mm to predict mortality which was higher than the cut-off point in our study which was 5.75 mm.

Correspondingly, in Komut et al. study [19], ONSD measurement with a cut-off point of 5.3 mm was able to detect non-traumatic brain injury with 70% sensitivity, 74% specificity, and an area of 0.728 under the ROC curve. However, the type of brain lesions was not restricted to ischemic lesions and the cutoff point brain injury predictions were less sensitive and specific than in our study. Comparable results were obtained by Aduayi et al., in which a 5.2 mm cut-off point with 81.2% sensitivity and 100% specificity was able to differentiate brain lesions in brain CT scan [20].

Regarding the etiology of stroke, the current study showed that ONSD predicts the severity of ischemic stroke independently of stroke type which means that the occurrence of ischemic events is associated with changes in the thickness of the optic nerve sheath regardless of the stroke mechanisms. The same concept is adaptable for the side of the brain being affected. Likewise, our results were extensible to patients in all age groups. Females, however, show a higher increase of ONSD in acute ischemic stroke.

With regards to the cut-off points utilized, extents of 5.65 mm for right ONSD (100% sensitivity and 86% specificity) and 5.75 mm for left ONSD (100% sensitivity and 88% specificity) were used to predict the severity of stroke. Previous studies have revealed different measures of ONSD in Asian and Western countries for evaluating intracranial pressure [12]. Indeed, predicting ischemic stroke based on ONSD may require introducing different cut-off points in different communities and geographical areas. Hence such studies on each ethnicity are necessary to reach a suitable global cut-off point range for each race.

Ultrasound can act as a practical surrogate modality along or before routine neuroimaging studies, for detecting the severity of acute ischemic stroke. This would help clinicians in decision-making and would result in prompt management which ultimately favors the patients’ outcome. Ultrasound is a non-invasive technique that is not expensive and its applicability shall be highlighted in more remote or crowded centers where other image modalities such as Brain CT scan or brain MRI are not easily accessible. In extreme situations, perhaps ultrasound might substitute the routine neuroimaging. Therefore, we recommend further studies in this area to confirm the association of ONSD and clinical stroke severity measured by NIHSS corresponded with neuroimaging findings.

The results of this study indicate a predictive value in the ultrasonographic evaluation of optic nerve sheath diameter (ONSD) for assessing the severity of acute ischemic stroke. The strong correlation we observed between ONSD and both initial NIHSS and clinical recovery (ΔNIHSS) can be explained by the pathophysiology of ischemic brain injury. Acute ischemia triggers a cascade beginning with cytotoxic edema, where ion pump failure leads to cellular swelling. This can progress to vasogenic edema as the blood-brain barrier disrupts, allowing fluid and proteins to leak into the interstitial space [21, 22]. In large-territory infarcts, particularly those resulting from anterior circulation large vessel occlusions (LVO), this edema can lead to significant mass effect and a rise in intracranial pressure (ICP) [23]. The ONSD, as a known surrogate for ICP, increases as the subarachnoid space around the optic nerve distends in response to this pressure transmission from the cranial cavity.

Our measurements were taken within hours of symptom onset, which is a critical strength for early triage but also frames an important question of time dependency. Malignant MCA infarction, the most severe form of ischemic edema, typically peaks 2–5 days after onset [24]. Therefore, an elevated ONSD at admission likely identifies patients with such a large initial core of infarction and such profound early cytotoxic edema that they are on a trajectory toward malignant edema and significantly elevated ICP. This aligns with our finding that a high baseline ONSD predicts poor recovery.

From a clinical perspective, this raises the provocative possibility that ONSD could serve as an early, bedside biomarker to help identify patients at highest risk for developing malignant edema. This could be particularly impactful for the early selection of patients who may be candidates for life-saving decompressive hemicraniectomy [25]. While current selection relies heavily on clinical exam and follow-up neuroimaging, an admission ONSD above a certain threshold could provide an immediate, objective data point to flag these high-risk individuals, potentially triggering closer monitoring and expedited surgical evaluation.

Our study was not powered to perform a sub-analysis specifically on LVO strokes, and we did not systematically collect data on subsequent hemicraniectomy. This is a recognized limitation and a vital direction for future research. Prospective studies are needed to validate ONSD cut-offs specifically in an LVO population and to determine its predictive value for the subsequent development of malignant edema requiring surgical intervention.

## Conclusion

Ultrasound-measured ONSD at admission is a strong, non-invasive estimator of both initial severity and recovery potential in acute ischemic stroke, demonstrating high sensitivity and specificity. This positions it as a promising dual-purpose tool to aid clinical decision-making alongside routine imaging. To refine its application, future studies should directly compare these ONSD cut-offs with those from hemorrhagic stroke patients to establish its subtype-specific utility in acute stroke management.

## Data Availability

Authors declare the all data and materials as well as software application or custom code support their published claims and comply with field standards.

## Abbreviations

ONSD: Optic nerve sheath diameter
ICP: Increased intracranial pressure
CPP: Cerebral perfusion pressure
NIHSS: National Institutes of Health Stroke Scale
CT: Computerized tomography
MRI: Magnetic resonance imaging
USG: Ultrasonography
